# Compassionate Conservation: Exploring the Lives of African Wild Dogs (*Lycaon pictus*) in Botswana

**DOI:** 10.3390/ani9010016

**Published:** 2019-01-07

**Authors:** Valli-Laurente Fraser-Celin, Alice J. Hovorka

**Affiliations:** 1 Department of Community Health Sciences, University of Calgary, Calgary, AB T2N 4Z6, Canada; 2 Faculty of Environmental Studies, York University, Toronto, ON M3J 1P3, Canada; ahovorka@yorku.ca

**Keywords:** compassionate conservation, animal geography, animal subjectivity, animal agency, responsible anthropomorphism, wildlife conservation, animal welfare, African wild dogs (*Lycaon pictus*), Botswana

## Abstract

**Simple Summary:**

This paper argues that animals should be positioned as subjects in research and scholarship to further develop compassionate conservation, a new field that aims to bridge conservation biology and animal welfare science. Animals can be treated as subjects by attending to their lived experiences and by recognizing their capacity to act. This paper merges interviews, blog posts, biological research, and observations to position African wild dogs as subjects in conservation research and scholarship using responsible anthropomorphism. It presents wild dogs as thinking, feeling, and sentient animals who have agency (capacity to act), and whose welfare is negatively affected by habitat loss and conflict with farmers. By positioning wild dogs as subjects, we can develop an ethical starting point for a more compassionate conservation. This ‘enriched’ scholarship allows us to more fully appreciate the complex lives of wildlife, their circumstances, and their experiences.

**Abstract:**

This paper argues for a more compassionate conservation by positioning animals as subjects in research and scholarship. Compassionate conservation is a multidisciplinary field of study that broadly attends to the ethical dimensions of conservation by merging conservation biology and animal welfare science. However, animal geography is rarely discussed in the compassionate conservation scholarship despite sharing similar tenets. This paper argues that responsible anthropomorphism and animal geography concepts of animal subjectivity (lived experiences) and agency (capacity to act) positions African wild dogs (*Lycaon pictus*) as subjects in conservation research and scholarship. It merges biological research, public communication, and interview and participant observation data to present wild dogs as thinking, feeling, self-conscious animals with agency, and whose welfare is negatively affected in human-dominated landscapes in Botswana. This paper argues for more attention to be paid to animal subjectivity and agency to foster more compassionate relations with wildlife. It argues that positioning animals as subjects in research and scholarship is an ethical starting point for moving compassionate conservation forward. This ‘enriched’ scholarly approach moves us closer to appreciating the lives of wildlife and the complexity of their circumstances and experiences.

## 1. Introduction

Compassionate conservation is an emerging multidisciplinary field that broadly focuses on the ethical dimensions of conservation; compassion, in the field of compassionate conservation, is defined as “empathy in humans for non-human animals and a drive to alleviate suffering” [1] (p. 270) while ethics are defined as “how we should live our lives, what ends we should seek, and what means we should use in pursuit of our ends” [2] (p. 281). In particular, compassionate conservation is concerned with wildlife welfare, and aims to bridge conservation biology and animal welfare science, two disciplines that have historically been seen as separate [3,4]. There is a growing interest in the field to produce “creative ethical” dialogue when attending to the welfare of wildlife [5]. To contribute to this dialogue and to the growing field of compassionate conservation, we argue that we must pay attention to animals’ inner lives and agency in conservation scholarship and practice [6]. We use a case study of human perceptions of and experiences with African wild dogs (*Lycaon pictus*) in Botswana to position animals as subjects by considering the concepts of subjectivity, agency, and welfare using responsible anthropomorphism. To consider these concepts, we bring together data from biological studies and wild dog researchers’ public communications (i.e., blog posts and reports) with animal geography methodologies (responsible anthropomorphism, semi-structured interviews, and participant observation). Positioning animals as subjects extends theoretical and conceptual underpinnings of compassionate conservation, while at the same time contributes a multidisciplinary approach (i.e., wildlife welfare, conservation biology, and animal welfare merged with animal geography concepts and methodologies) to understanding the lives of animals more fully. We also demonstrate that we must consider the lives of wild dogs in the political-economic and socio-cultural context of Botswana alongside human-wild dog dynamics. As such, we demonstrate that we cannot fully understand wild dogs through biology alone. Rather, to fully understand wild dogs, we need to consider their influence, their emotions, their individuality, and their welfare; by considering these characteristics, we demonstrate that wild dogs are thinking, feeling, and sentient beings who shape their own lives, their circumstances, and the lives of others.

The remainder of this paper is organized as follows: first, we discuss compassionate conservation, its central tenets, research, key debates, and gaps in the scholarship. We also present a brief overview of the fields of conservation biology and animal welfare and how these disciplines differ and share similar tenets. We give a brief overview of compassionate conservation’s roots in conservation biology and ethics, and then discuss the concept of animal sentience. Second, we discuss the sub-discipline of animal geography. Third, we discuss animal agency and subjectivity in the context of compassionate conservation and animal geography. Fourth, we present responsible anthropomorphism as a methodological framework that brings into focus the experiences and circumstances of animals. We contend that we can make preliminary explorations about what animals think and feel through shared mental capacities and experiences. We then provide details on African wild dogs in Botswana and data collection and analysis. Finally, we present wild dog subjectivity, agency, and welfare through different data sources that explore the lives of wild dogs in Botswana. We conclude by arguing that a next step in compassionate conservation is to pay attention to these elements in order to demonstrate that the lives of animals (wild dogs in this case) are complex and nuanced, and shaped by broader social structures and their interactions with humans.

## 2. Compassionate Conservation

Compassionate conservation was conceptually developed by the Born Free USA foundation in partnership with the Wildlife Conservation Research Unit (WildCru) at Oxford University. The field of compassionate conservation is concerned with reconciling the long-standing disconnect between conservation biology and animal welfare [7]. While the contemporary fields of conservation biology and animal welfare developed alongside each other, they have largely remained politically and scientifically separate. Conservation biology developed as a science focused on preventing extinction, and the conservation movement began with protecting populations and ecological systems from exploitation. Today, conservation biology research and practices remain broadly focused on species, populations, and ecosystem preservation [4,8,9]. While conservation biology has its roots in population ecology, understanding individual animal behavior can potentially increase understandings around animals’ capacities to survive in fragmented habitats, population responses to disturbance and exploitation, and population monitoring and modeling, among other studies of species and populations [10]. Modern conservationists use sophisticated behavioral analyses of animals they aim to conserve such as movement and spatial use, reproductive behavior, social organization, foraging and vigilance, and interactions with humans and other animals [11]. These behavioral analyses can, for example, provide early warnings of habitat degradation and population declines and can be used to monitor outcomes of conservation management programs or evaluate their success at the early stages [11]. Compassionate conservation’s roots in conservation biology are most evident in its focus on protecting wildlife [5]. Conservation biology has evolved to reduce harm to wildlife and other animals, as well as adopting compassionate ways of minimizing conflicts between humans and animals (e.g., fencing to deter predation on livestock and conservation education programs); as such, compassionate conservation challenges conservation biologists and other decision-makers to have clear values and objectives when the lives of (individual) animals are concerned [12].

Animal welfare science, on the other hand, emerged in response to the cruelty and neglect of domestic and captive animals, resulting in numerous laws banning animal cruelty in agriculture, sports, work, and research. Sentience, defined as the capacity to experience positive and negative affective states, is central. The capacity for animals to suffer was the common-sense perception among communities interacting daily with animals as early as the Renaissance. However, sentience became of interest to biologists only in the last 40 years and quickly became a central concept with two theoretical schools of thought emerging with regard to its definition and measurement within contemporary animal welfare science [13]. Initially concerned with physiological stress during the 1960s and 1970s, more in-depth welfare studies resulted in theoretical debates and schools of thought around whether animal welfare was associated with animals’ biological functioning or feelings. While the “biological functioning school” focuses on the absence of stress, the ability to cope, and the animal’s biological needs, the “feelings school” associates sentience with suffering and pleasure [14]. While animal welfare science continues to focus predominately on domestic animals, some animal welfarists are becoming concerned with conservation practices that trade-off one species to benefit another, or practices that trade-off individuals to benefit the species [8,15].

Ethically and politically, compassionate conservation adopts a “first do no harm” approach [5,7]. While conservation biology and animal welfare science have emphasized utilitarian and economic ethics and values, compassionate conservation foregrounds intrinsic and aesthetic values [15]. At the same time, compassionate conservation extends into social justice (“the fair treatment of others judged according to three principles: equality, need, and desert (noun form of deserve)” [16] (p. 23)). This social justice approach, as it applies to conservation [16,17,18], must also include nonhuman animals and should be guided by a non-anthropocentric principle with special concern for nonhuman animals’ interests alongside humans’ [16,19]. In particular, compassionate conservation attempts to tackle the notion that “conservation biology and its ethics remain confused about whether we have responsibilities to individual organisms” [17] (p. 134). By bringing conservation and animal welfare together, compassionate conservationists are tackling the long-standing debate that the two disciplines should be regarded as separate (see [4]). Despite the two fields’ different topics of interest, research, and scale, conservation biology and animal welfare science both inherently share a social concern for animals [8]. With humans and wildlife increasingly sharing spaces, the same human activities that cause wildlife extinction also cause suffering and trauma in individual wild animals, an important argument for bringing conservation and animal welfare into conversation [9].

In 2010, the first compassionate conservation conference was held, bringing together scholars and practitioners from disciplines across the natural and social sciences and humanities to discuss animal welfare issues in conservation. The conference established compassionate conservation as a growing multidisciplinary field. Compassionate conservation involves cross-disciplinary research conducted predominately by conservation biologists concerned with wildlife welfare [2,5,9,20,21] and animal welfarists concerned with the implications of conservation practices on the welfare of wildlife [7,8,14,22,23]. The field encompasses work situated in architecture and planning [24], veterinary science [25], and conservation psychology and economics [3,26], among other disciplines from the social sciences and humanities [27,28,29,30,31]. Research to date has broadly focused on wildlife welfare in human-dominated landscapes [7,8,9,22,32], wildlife captivity, rehabilitation, and translocation [21,22], and conservation ethics [2,32]. For example, Paquet and Darimont [9] discussed the various ways wildlife welfare is compromised in human-dominated landscapes, such as noise pollution and habitat fragmentation; Ramp [21] described and discussed the ethical dilemma of the wild kangaroo harvest in Australia; and Moore, Wihermanto and Nekaris [21] examined viable options that address the both welfare and conservation goals of wildlife in rescue centers.

Compassionate conservationists are therefore laying the groundwork for future studies to begin taking wildlife welfare more seriously in both conservation and animal welfare research, practice, and scholarship. Moreover, the compassionate conservation field’s inclusion of various disciplines opens up space for more interdisciplinary and collaborative research and practice. Collaboration among individuals who can bring different theoretical, conceptual, and methodological approaches is regarded as a step toward improving conservation practices by addressing conservation problems that are interdisciplinary in nature, such as wildlife welfare [9,33,34]. However, despite sharing similar important tenets, animal geography, is not yet discussed and rarely foregrounded in the compassionate conservation scholarship (however, see [15,19]).

## 3. Animal Geography

Research in the natural sciences often takes an objective or neutral stance on its subjects of study. In the past (and still sometimes today), the natural sciences have portrayed animal behavior as predetermined by instincts and genes [35]. Not only does this avoid considering animals as subjects, but it also fails to consider animals as individuals with their own lives and experiences [27,36], an aspect that conservation biology generally does not focus on [17]. Consequently, Crist argued that a denial of animal minds leads more broadly to environmental destruction by humans: “As animals became successfully represented in dominant discourses as devoid of agency and experiential perspective—thereby becoming construable as a means for human ends—a fortiori the (apparently) nonsentient domains of forests, rivers, meadows, oceans, deserts, and mountains (in fact, of any landscape or seascape) were made accessible to the human race without accountability or restriction” [27] (p. 46). As such, there have been several calls by critical animal scholars, including animal geographers, to attend to the inner lives, experiences, and worldviews of animals [37,38]. Animal geographers attempt to highlight animals’ roles in human societies and identities, and in particular, their subjectivities, and their agency as beings with inner thought and intentionality [39,40,41].

However, in this paper, we argue that conservation biologists’ scientific scholarship and public communications are indeed producing insights into the lives of animals; animal geographers have recently begun to recognize this and have made calls for more collaboration with natural scientists [37,42,43]. Bringing together different perspectives of animals may therefore be more fruitful for understanding the inner lives of animals in a non-anthropocentric way [17,37,43].

Compassionate conservation and animal geography, a sub-discipline of human geography, share two important foundational tenets: (1) both bodies of scholarship developed as a response to the ethical and political responsibilities we hold toward the animals we share our world with [42,44]; and (2) both bodies of scholarship seek to ensure that (individual) animals’ needs are not simply ignored or unthinkingly placed below humans’ needs [7,15,44]. However, animal geography also moves beyond these anthropocentric concerns and attempts to understand the lives of animals in and of themselves, not only as individuals, but as beings who have lived experiences and agency [37,38,43,45,46]. Animal geography is therefore well-positioned to contribute to extending the field of compassionate conservation by highlighting these aspects.

By engaging with animal geography methodologies such as responsible anthropomorphism and highlighting concepts of animal subjectivity and agency, animals can be positioned as subjects by acknowledging that they suffer, experience pleasure, and make decisions that affect their own lives and the lives of others. We also discuss animal welfare and how it is represented in conservation biology scholarship, biologists’ public communications, and animal geography examinations (interviews and participant observation) of the lives of wild dogs in Botswana. This multidisciplinary approach enables us to better understand animals as “minded subjects” [47] by positioning them as “thinking, feeling, beings, with lives worthy of consideration” [48] (p. 119). Moreover, while animal welfare science focuses on animal sentience, with studies examining animals’ physical and emotional responses to environmental conditions; animal geographers focus on animal welfare by exploring the politics around the treatment of animals. For example, animal geographers have examined socio-spatial and political-economic structures and processes that may affect animals’ circumstances; they have explored the wildlife trade, environmental politics of caring for wildlife, the politics around habitat destruction, and human-animal relations of power [45,46,47,48,49,50,51].

Building on the aforementioned shared tenets, we argue that engaging with animal geography concepts of animal subjectivity and agency will: (1) integrate animal geography more broadly into compassionate conservation scholarship; and (2) position animals as subjects in compassionate conservation scholarship. In biological research and scholarship, animals are most often positioned as homogenous objects or units of analysis [36,52,53,54]. For fear of stepping into the realm of anthropomorphism, animals are often portrayed as “without individual character, knowledge, subjectivity or experience” [55] (p. 196). Portraying animals as sentient, thinking, feeling beings who have the capacity to act, and affect the lives of others would extend the compassionate conservation scholarship.

## 4. Animal Subjectivity and Agency

Subjectivity, in this case, refers to how animals live in the world and what they experience; subjectivity also portrays animals as thinking, feeling, sentient, and self-conscious individuals [37,45,47,56]. One important conversation in animal geography concerning animal subjectivity centers around how we can understand the experiences of other animals. Animal geographers such as Bear [37], Buller [52], Hodgetts and Lorimer [42] and Hovorka [43] contend that greater engagement with disciplines such as the animal sciences, ethology, and biology, which have the tools and in-depth knowledge of animal behavior, is needed. For example, Geiger and Hovorka used animal welfare methods to consider donkey subjectivity as a “lived experience of the body” [45] (p. 1102) by assessing donkeys’ physical and emotional experiences and welfare. The authors considered the lives of donkey lives through their daily activities, relationships with their owners, and body conditions, finding that most donkeys in Botswana experience trauma and hardship.

Agency, in this case, refers to the capacity to influence one’s life and the lives of others, to exert power, and to achieve goals [57,58]. Animal agency is defined in various ways in animal geography. Dempsey [46], Van Patter and Hovorka [38], and Notzke [59] argued that agency is not an inherent or fixed trait; rather it is made through relationships, conflict, negotiations, and alliances with other animals, humans, and the environment. Philo [54] took another approach by discussing agency as reactive to human spatial orderings; animals enact their agency when they resist or transgress the material and conceptual spaces allocated to them by humans. For example, Power [60] described how possums engage in “border ruptures” by leaving the “wild” and inhabiting the walls and ceiling cavities of people’s homes in Australia, contributing to humans’ feelings of either anxiety or homeyness within their households. Rutherford [58] described agency as being enacted both directly and indirectly; it is enacted directly when animals evade humans and indirectly when policies and laws that affect humans or landscapes are written because of animals. Meanwhile, Lorimer [61], Hovorka [62], Dempsey [46], and Notzke [59] focused on the role of animals’ aesthetic and ecological characteristics in shaping their own lives through their interactions with humans and their environments.

Including animal subjectivities and agency into compassionate conservation scholarship means that we may be able to make some preliminary explorations about how wild dogs may be feeling and thinking, and their affect in the world, positioning them as subjects in research rather than objects of research. This would provide fuller accounts of their lives and welfare in Botswana by foregrounding their sentience, as well as their “conscious, emotional, cognitive and creative inner life” [43] (p. 4).

## 5. Responsible Anthropomorphism

How do we position animals as subjects in conservation scholarship? Animal geographers are interested in exploring “what an animal worldview may be, how an animal may wish to represent itself, and how we may come to know these expressions” [43] (p. 2). Exploring an animal’s point of view is a matter of understanding and representing different ways of knowing the world [35,63]. However, how do we come to know this other way of knowing? How do we accurately represent them? Anthropomorphism, or “the use of human characteristics and mental states to describe, explain, or anticipate animal behavior”, is one way humans attempt to understand animal worldviews and expressions [35,64,65]. Since the 17th century, scientists have discouraged such anthropomorphic thinking because they believed that differentiating between animals and humans would “improve critical thinking about animals’ behavior” [66]. However, in more recent times, some scholars argue that humans can achieve mutual understanding and plausible empathy with animals based on shared sociality, intelligence, and sentience [35,44,63]; this is notably reflected in the works of primate ethologists Jane Goodall, Dian Fossey, and Birute Galdika [55].

As humans, we empathize everyday with other humans to understand them, and, as researchers, we represent our subjects’ perspectives through our own personal lens. It is therefore only a “short empathetic step” to imagine the world through the eyes of animals [67] (p. 130). Anthropomorphizing occurs because people develop an empathetic understanding of how animals behave through shared experiences and mental capacities, developing understandings of animals based on co-relationality [35,44,48,68].

Conservationists often use anthropomorphism as a tool to promote target species by developing public empathy and conservation awareness [69]. Within scientific scholarship, however, researchers are expected to remain detached from the lives of animals and to use language that does not assume animal mental states or emotions [35,36,55]. While conservationists use methods such as observation, camera trapping, tagging, and tracking to understand and analyze the daily habits and lives of animals [36,42], anthropomorphizing occurs through personal experiences, anecdotes, stories, myths, and lore [63]. Bekoff [63] argued that these data sources are also important because there are many correct ways to describe or explain what animals feel or do. Similarly, Russell [70] asserted that stories help us to imagine an animal’s perspective and experiences, while Rollin [71] contends that plausibility and common sense assumes that animals, similar to humans, feel emotions such as pain, fear, curiosity, and other mental states.

Anthropomorphizing, however, if done irresponsibly, may put unrealistic expectations on animals to behave in human-like ways and fail to consider that animals most likely see the world in a very different way than we do [40,72,73]. Further, an “irresponsible anthropomorphism” may be misleading in that it may characterize animals in a fashion that may not accurately represent the human behavior or emotion it is attempting to reflect; Beck [66] illustrated this point through a critique of Elizabeth Marshall Thomas’ likening the relationship between two dogs as a “marriage” in her book The Hidden Lives of Dogs, whereby not only are the legal and social implications of marriage ignored, but the cultural differences as well, where not all cultures engage in marriage rituals.

A responsible anthropomorphism relies on the stories, anecdotes, and narratives of individuals who spend time with animals, such as biologists, naturalists, hunters, and trackers. For example, Lorimer [74] explored of the embodied skills and emotions of scientists involved in corncrake counting in the UK. The scientists “tune-in” to the corncrake through scientific and folk knowledge of corncrake behavior and ecology. They attempt to embody the birds’ behavior in order to track them in their natural habitat, as Lorimer explained: “In this wild ethology they immerse themselves in the field and feel for the bird” [74] (p. 384).

Timberlake and Delameter proposed that to understand animal behavior, researchers “not only need to put themselves in the subject’s shoes, they need to wear them—walk, watch, hear, and act like the subject” [75] (p. 39). Feminist scholars in particular have been exploring and promoting embodied experiences and approaches to research within animal studies by focusing on the body as a unit of analysis, on feminist ethics of care, and on empathic relations and understanding [76]. Feminist concepts of performativity and embodiment, or embodied knowing, have been applied to explore how animal bodies perform and reproduce power relations, experiences, and place-based dynamics and context [45,48,76,77,78]. Geiger and Hovorka [45] also used the concept of performativity (alongside animal welfare methods) to explore the relationships between donkey bodies, place-based power relations, and donkey identity in Botswana; exploring animal experiences through embodied practices means “sharing *across* boundaries of bodily responses” [77] (p. 154, emphasis in original). Ultimately, a responsible anthropomorphism enables humans to recognize (vertebrate) animals as interactive subjects in the world who share commonalities with humans yet, at the same time, have their own unique experiences, thoughts, and individuality.

## 6. Learning about Botswana’s Wild Dogs

**Figure 1:**
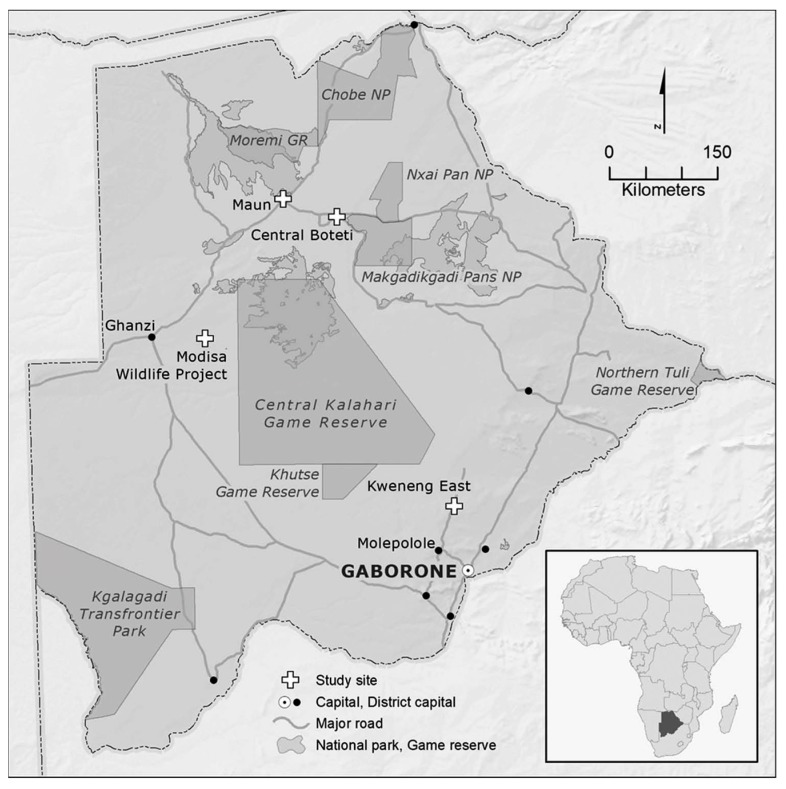
Study Areas in Botswana, Africa.

Botswana is a semi-arid, land-locked country in southern Africa (Figure 1) and home to some of the most diverse and largest populations of wildlife on the continent. Predominately unfenced, wildlife roam freely across protected area and game reserve borders into human settlements; in Botswana, humans and wildlife share the landscape. The country’s socio-economic reliance on cattle, which imbues men with social standing and acts as a bank account and source of food security, engenders incidents of human-large carnivore conflicts, where large carnivores predate on cattle and farmers engage in retaliatory killing and injuring of ‘problem’ carnivores. At one time, African wild dogs roamed freely across the African continent. Today, they have lost most of the habitat they historically occupied due to human encroachment and subsequent persecution, disease, and road mortality. Effectively eradicated from north and west Africa and largely reduced in northeast and central Africa, southern Africa and the southern part of east Africa are home to the remaining 6600 wild dogs on the continent [79]. Botswana is home to approximately 1310 wild dogs [80].

To understand wild dogs and their lives, we used multiple data sources including biological studies, public communications, interviews, and participant observation. Together, these data sources explore the lives of wild dogs in Botswana beyond only biological studies, including the way they live apart from humans; their interactions with each other, humans, and other animals; their emotions; aspects of their welfare; and their lives in Botswana’s social context. We followed ethical guidelines for involvement of human participants (Research Permit #EWT 8/36/4 XXII [3]) and animal subjects in accordance by those provided and approved by the Ministry of Wildlife, Environment and Tourism (Government of Botswana), as well as the University of Guelph Research Ethics Board.

We gathered documents such as biological studies, publicly available blog posts, and reports on wild dogs in Botswana. Biological studies were written by conservationists who study wild dogs in Botswana. Conservation biologists explore the lives of wild dogs in Botswana using methods and tools such as GPS activities and location data, VHF and aerial tracking, sightings, photographs, videotapes, direct observations, historical data, general health recordings (e.g., blood samples and body measurements), as well as questionnaires and analyses of Problem Animal Control registers. Blog posts and reports were written by biologists from the Botswana Predator Conservation Trust (BPCT) and the Kalahari Research and Conservation (KRC) group. BPCT and KRC are the two main groups conducting research on wild dogs in Botswana; together they provide data on wild dogs across almost the entire country (BPCT conducts research in northern Botswana and the Ghanzi area and KRC conducts research on wild dogs in the eastern Kalahari region and in the Central Kalahari Game Reserve). Blog posts and reports are considered to be accounts of both scientists’ field work and what animals do and why. Rees described these accounts as “part adventure story, part autobiography, part textbook” [36] (p. 7). Blog posts and reports were therefore useful for understanding wild dog subjectivity, agency, and welfare from a different perspective.

Finally, researching human-wild dog conflict and conservation in Botswana put us in a unique position to hear narratives, stories, and anecdotes about wild dogs, and to observe wild dogs. We spent 10 months in Botswana, from May to July 2013 and again from February to July 2015. We conducted 110 interviews in four study sites (Central Boteti, Kweneng East, Maun, and at the Modisa Wildlife Project; Figure 1), where we interviewed individuals who had in-depth knowledge of and experiences with wild dogs, or who shared similar environments. Participants included individuals working in the agriculture, tourism, and conservation industries (e.g., cattle and game farmers, government officials, wild dog researchers, and safari guides among others). We also engaged in participant observation by immersing ourselves in the everyday context of living in Botswana by having informal conversations and exploring how people and wildlife live in the country, as well as observing free-ranging and captive wild dogs. We grounded the qualitative, thematic analysis in the animal geography and animal welfare scholarships. We used latent content analysis to analyze the data for passages that reflected animal subjectivity, agency, and welfare according to these bodies of scholarship.

## 7. Wild Dog Subjectivity, Agency, and Welfare

“Predators are at the forefront of a compassionate revolution in conservation”

[5] (p. 1481)

The following section positions wild dogs as subjects by paying attention to wild dog subjectivity, agency, and welfare in Botswana. It brings together the different ways natural and social scientists are exploring the lives of wild dogs in Botswana. By merging the conservation biology, public communication, interviews, and participant observation data sources, we demonstrate that the lives of wild dogs are complex, nuanced, and emotional. This study also reveals that we cannot understand the lives of wild dogs through biology alone; attention must be paid to human-wild dog dynamics and the broader context in which wild dogs live their lives. Through the following narratives, we present a depiction of the animals as the subjects who are truly “at the heart of the research process” [36] (p. 3).

### 7.1. Wild Dog Subjectivity

Wild dogs are thinking, feeling, self-conscious animals with personalities, moods, and strong social bonds. Lila, Kubu, Thuto, Dotski, Trinity, and Taryn; and Mula Pack, Tsau Hills Pack, and Mankwe Pack; these are some of the names conservation biologists in Botswana have given to individual wild dogs and wild dog packs. While these names do not appear in conservation biology scholarship, researchers use names to identify and engage with individuals. At the same time, naming implies individuality and personality [81]. Wild dog moods and emotions were discussed in blog posts and in interviews. Wild dogs were described as “playful”, “curious”, and “happy”, as one participant explained: “… their chittering when they get really excited, the squeaking noise that they make. You just know that they’re excited and that they’re happy, happy animals”. Our own interactions with wild dogs reminded us of experiences with domestic dogs; they exhibited similar behaviors such as curiosity, caution, excitement, and playfulness; they wag their tails and bark like domestic dogs. However, wild dogs were also described by participants as being “tricky”, “destructive”, and “cruel”. One participant explained: “Have you ever seen a wild dog kill? It is one of the most vicious things. They run it [prey animals] down, it’s tired, they virtually start to feed on it live, disembowel it.” While these latter characterizations may be critical of wild dogs, they do lend wild dogs the capacity to think, make decisions, and act [49].

Wild dogs are social animals with strong pack bonds who hunt, feed, and raise pups as a group [82,83,84]. Many participants likened them to a human family: “I like their spirit of being together, like a real family, together. We always see them together, how the mothers take care of the puppies […] It’s really serious care.” Participants also described them as altruistic: “they’re probably the best example in the animal kingdom that I can think of a social system that actually works on altruism.” In Botswana, wild dogs have been found to engage in group decision-making where certain pack members sneeze to make decisions around rallying (i.e., group departures). This illustrates that “specific behavioural mechanisms (here, sneezing) allow for negotiation (in effect, voting) that shapes decision-making in a wild, socially complex animal society” [85] (p. 284). Wild dogs also use scent-marking through urination to communicate the limits of their territory with other packs [86,87].

Life histories of wild dogs are shared through stories of wild dog dispersal and pack membership in BPCT blog posts: “The pack I spent most of my time with was the local Apoka Pack, in the area since 2013. Darius, an immigrant male of unknown origins and Seronera, a disperser from the extinct Mathews pack were—and still are—the Apoka pack dominants” and “… three of the older sub-ordinate males—known to us as Claudio, Stetson and Toque—were absent. It seemed that that day they had left their natal pack to try and establish one for themselves.” By sharing the life histories of wild dogs, we learn more detail about the individuals that make up the biological studies of wild dogs as a species. This brings us closer to compassionate conservation by considering the lives of individual animals within a species and demonstrates that “there can be variation between individuals and within an individual” [88] (p. S4).

### 7.2. Wild Dog Agency

Wild dogs shape their own lives, those of other animals and those of humans directly and indirectly, and exert power. Wild dogs enact their agency by crossing protected area borders and moving into human settlements where they prey on livestock animals and use human structures such as fences and roads [84,86,89,90,91,92,93]. Wild dogs learn about and understand the changing dynamics of their habitats and adapt to make use of different features to their advantage. These attributes demonstrate how wild dogs shape their own lives and address challenges posed to them such as the presence of and competition with lions and hyena, lack of wild prey, and presence of human settlements [84,89,90,91,92,93,94].

Biological studies and blog posts discuss wild dogs’ movements into human settlements as reactionary to habitat loss/fragmentation and the presence of other large carnivores. However, in doing so, wild dogs not only move out of a space that is allocated to them, but they also move out of the role humans assign to them—as wild animals who belong in protected areas [54,94] as one participant explained: “It would be better if it [wild dog] was fenced in to be separated from domestic animals because they destroy my livestock.” Through an animal geography lens, wild dogs do not only react to habitat loss, but they also work with their circumstances and adapt to their changing natural environments by moving into human settlements. Further, while biological scholarship discusses habitat through scent-marking and home ranges, their habitat is also their home. Establishing home ranges and territories through scent-marking and movements across the landscapes are actions that “coalesce in a narrative of getting to know a space, marking one’s territory, and making a place one’s home” [38] (p. 16). This positions wild dogs not as occupants of a space, but rather as inhabitants of Botswana’s landscapes [38].

In public communications, conservationists discussed individual wild dogs and how wild dogs affect them: “When you spend your time studying wild dogs, you cannot help but come to know them as individuals” (BPCT blog post) and “I had developed a special affection and a quite personal bond with those two individuals. It saddens me that my last memory of those inquisitive and adept individuals is of them rotting in the sun beside the buffalo fence, stark reminders of the challenges that predators face in man’s [sic] world” (BPCT blog post). Conservationists expressed excitement at seeing wild dogs alongside the challenges of finding such elusive animals; wild dog agency is apparent through this elusive nature and especially when they evade researchers: “We immediately followed them into the bush. Despite many obstacles, we managed to stick with the dogs for roughly 300 m. But then, suddenly, we lost them in a relatively open area” (BPCT blog post). The emotions and challenges wild dogs present are rarely discussed in the conservation scholarship, yet, through these blog posts, we gain a greater sense of wild dog agency through their capacity to affect biologists. These negotiations with the biologists also reveal wild dog agency when wild dogs evade or move away from researchers. In doing so, they are asserting their role in the research process [38,46,59].

Wild dogs also enact their agency not only through their negotiations with researchers but through their conflict with farmers as well. Many farmers were negatively affected by wild dogs and expressed feelings of hopelessness concerning wild dogs’ threat to livestock: “Due to destruction, they make me sad because they make people poor. Instead of more livestock, they decrease the numbers”. Some farmers changed their behaviors because of wild dogs: “If I’m alone I don’t go get my cows, I went alone before. I’m scared of walking alone. Every time I go out I make sure there is somebody or else I don’t go out […] If these cows have gone far into thick bush, I won’t go.” Moreover, because of their endangered status, wild dogs are listed as “protected game animals” in Botswana’s Wildlife Conservation and National Parks Act meaning they cannot be legally killed even if they pose a threat to livestock. Because of this policy, farmers often fear the government and the repercussions of injuring or killing a wild dog, as one farmer explained: “I’m afraid of being arrested”. Wild dog agency is therefore apparent both directly and indirectly in the ways they shape the lives of farmers through their actions and presence.

### 7.3. Wild Dog Welfare

While not explicitly discussed, wild dog physical and emotional welfare is affected by habitat loss and fragmentation, and conflict with farmers. Habitat loss and fragmentation were described as leading factors in long-term wild dog population viability and wild dog extinction [84,86,90,92,93]. According to Abrahms et al., “an ability to move through its landscape has fundamental consequences for both individual fitness (e.g., resource acquisition, survival) and long-term population persistence” [89] (p. 247). A blog post explains: “Of Africa’s prized large carnivore species, the endangered African wild dog needs the most space to survive and as a result has suffered the greatest impacts from the loss and fragmentation of its habitats”. Anthropogenic landscapes also result in disease transmission from domestic dogs such has rabies and canine distemper as well as road incidents [84].

Conflict with farmers significantly affects wild dog welfare. For example, in the three months following Gusset et al.’s [91] study of human-wild dog conflict in northern Botswana, 19 pups and six adults disappeared from the area, presumably shot. A blog post details the death of two wild dogs, Taryn and Trinity: “We wondered about snakebite, but that didn’t make sense, the two dogs dying together. […] upon returning to the carcasses, we found 12 dead and dying vultures; white-backed, white-headed and lappet-faced. We didn’t need to do any tests. The vultures’ deaths proved poison was involved” and a report by the KRC details the persecution of wild dogs in the Kalahari region: “We met with farmers whom have confessed to shooting the missing pack members and nine pups in the last few weeks”. Moreover, with wild dogs being persecuted, packs may become disbanded; one participant explained that losing pack members can be devastating for wild dogs, and that a lone wild dog would “die of a loneliness of heart”. Given the social nature of wild dogs and that animals are sentient, feel emotions, and have social knowledge [35], they may indeed feel loneliness and sadness when pack members are killed. These stories of wild dog deaths show the stark reality of what wild dogs experience, demonstrating that their lives in human-dominated landscapes are indeed “bloodier than we might like to think” [44] (p. 646).

States of suffering such as pain, fear, frustration and social deprivation have been elucidated in mammals and birds for at least 30 years [12,13,95,96,97]. Conservationists now accept that animals can experience fear, pain and distress as well as psychological and emotional trauma [9,98]. Wild dogs must therefore feel a certain amount of fear, stress, and other negative mental, emotional, and physical states in these conflict situations.

## 8. Wild Dog Subjects

“… scientists are privy to a rare and precious opportunity when we come to know intimately nonhuman animals living in their own worlds. We have a responsibility to these animals to show other people who they really are—sentient beings who matter to one another, living lives as full of drama, and emotion and poetry as our own”

[99] (p. xv)

This paper argues that positioning animals as subjects in conservation research and practice is a “next step” in compassionate conservation. Merging biological studies and public communications (blogs and reports) with animal geography (attention to subjectivity and agency and responsible anthropomorphism) produces a more-holistic account of the lives of animals in conservation research, science, and scholarship. While conservation biologists may not use the same language and methodologies as animal geographers, they do indeed discuss animal subjectivity and agency in their research and scholarship, and they also present animals as subjects in their informal, public communication writings by giving them names, by detailing their moods, and by describing how wild dogs affect them. Conservation biology perspectives provide insights into wild dogs’ life histories, daily habits, moods, behavior, and their lives apart from humans. Animal geography builds upon these biological studies and public communications by exploring wild dog thoughts and feelings through a responsible anthropomorphism, relying on interviews with people who know wild dogs and their environments, participant observations, and shared mental capacities. Merging these data sources portrays wild dogs as thinking, feeling, sentient beings who have agency, whose welfare is negatively affected in human-dominated landscapes, and whose lives are shaped by Botswana’s political-economic and socio-cultural context, namely, the attachment to and role of cattle in the country. This demonstrates that the lives of wild dogs are complex, nuanced, and emotional, and that we cannot study the lives of animals through biology alone.

Responsible anthropomorphism can act as a methodological bridge between portrayals of animals-as-objects and animals-as-subjects within conservation. By integrating biological studies with other data sources, we can engage in a compassionate conservation that attends to animals’ welfare and inner lives more robustly by recognizing their subjectivity and agency. This multidisciplinary approach therefore elicits more “holistic and meaningful insights into the lives of animals” [43] (p. 7). If this type of compassionate conservation was implemented more broadly within conservation, it would portray animals as thinking, feeling, and agential beings, and would consider how their welfare is tied to their subjectivity and agency. If science informs conservation policy, perhaps we need a science that attends to wildlife in a more compassionate way; a conservation science that “prompts us to see target species not as animals that can be directly managed, but as creatures with an agency of their own” [6] (p. 234).

## 9. Conclusions

Wildlife are suffering because of human activities. The new field of compassionate conservation aims to address this suffering by bridging conservation biology and animal welfare. We argue that positioning animals as subjects in research and scholarship is an ethical starting point for moving compassionate conservation forward. This ‘enriched’ scholarly approach moves us closer to appreciating the lives of wildlife and the complexity of their circumstances and experiences. From this starting point, compassionate conservation practice can consider the lives and welfare of individuals, how humans intricately affect them and the broader social context in which wildlife live. Conservation practices that consider wildlife subjectivity and agency can (re)include ethics, values, and morals within conservation decision-making practices and policies.

Portrayals of animal subjects, their emotional lives, as well as their relationships with researchers can raise public awareness, because “The stronger the sense of kinship that can be created in the mind of the reader, the greater the likelihood that a sense of shared responsibility and of mutual participation in a moral community will also develop” [36] (p. 15). Conservationists and animal welfarists who wish to further engage with compassionate conservation may begin to include anecdotes and descriptive narratives of the lives of wildlife in their scholarship. They may also wish to engage in more interdisciplinary work with social scientists, for example animal geographers and feminist scholars, who have different philosophical, theoretical, conceptual, and methodological approaches to getting to know animals; similarly, animal geographers and other social scientists may wish to work with biologists who have access to and insights into the inner lives and daily habits of animals apart from humans. Through more multidisciplinary engagements to understanding the lives of animals more vividly and holistically, compassionate conservation scholarship can treat animals as “characters, as individuals with lives, feelings, histories and motives of their own” [36] (p. 883).
